# Timing of first antenatal care attendance and associated factors among pregnant women in public health institutions of Axum town, Tigray, Ethiopia, 2017: a mixed design study

**DOI:** 10.1186/s12884-019-2490-5

**Published:** 2019-09-18

**Authors:** Berhanu Gebresilassie, Tilahun Belete, Weyzer Tilahun, Betell Berhane, Senait Gebresilassie

**Affiliations:** 1 Department of Midwifery, College of Medicine and Health Sciences, Adigrat University, Adigrat, Ethiopia; 2 Department of Nursing, College of Health Sciences, Mekelle University, Mekelle, Ethiopia

**Keywords:** Ante natal care, Timing, Pregnant women, Axum

## Abstract

**Background:**

Timely initiation of antenatal care can avoid pregnancy related problems and save lives of mothers and babies. In developing nations, however, only half of the pregnant mothers receive the recommended number of antenatal care visits, and start late in their pregnancy. Thus, the study was conducted to assess the magnitude of timely initiation of antenatal care and factors associated with the timing of antenatal care attendance in Axum in which studies regarding this issue are lacking.

**Methods:**

An institution based cross-sectional study mixed with qualitative approach was conducted. A total of 386 pregnant women were selected using systematic sampling technique for the quantitative study. In addition, 18 participants were selected purposively for the qualitative part. The quantitative data were collected using structured interviewer administered questionnaire while the qualitative data were collected using an open-ended interview guide. Quantitative data were analyzed using SPSS version 22 and the qualitative data were analyzed using Atlas software. Multi-variable logistic regression was used to control the effect of confounders.

**Results:**

The magnitude of timely attendance of antenatal care was 27.5% (95% CI: 23–32%). Unintended pregnancy (AOR = 2.87; CI 95%: 1.23–6.70), maternal knowledge (AOR = 2.75; CI 95%: 1.07–7.03), educational status of the women (AOR = 2.62; CI 95%: 1.21–5.64), perceived timing of antenatal care (AOR = 3.45; CI 95%: 1.61–7.36), problem in current pregnancy (AOR = 3.56; CI 95%: 1.52–8.48) and advice from significant others (AOR =2.33; CI 95%: 1.10–4.94) were found significantly associated with timely booking of antenatal care.

**Conclusion:**

The magnitude of timely attendance of antenatal care is low. Educational status, maternal knowledge, unintended pregnancy, problem in current pregnancy, perceived timing of antenatal care, and advise from significant others were the significant factors for timing of antenatal care. Therefore more effort should be done to increase the knowledge of mothers about importance of antenatal care and timely ante natal care booking.

## Background

First timing of antenatal care (ANC) attendance is defined as the first-time pregnant women come to antenatal clinics to get care from health care professionals. It is used to know the health status of the mothers and the fetus, to estimate the gestational age (GA) and expected date of delivery (EDD) and to initiate plans for next follow ups. World Health Organization (WHO) recommends that pregnant women in developing countries to get at least four ANC visits, and to initiate early ANC follow up. The 1st ANC timing to be before 16 weeks of gestational age, 2nd visit between 24 and 28 weeks of gestation age, 3rd visit between 30 and 32 weeks gestational age, and the 4th visit between 36 and 38 weeks of gestational age. Timely initiation of ANC is crucial for early detection of pregnancy related problems and adverse pregnancy outcomes like low birth weight, still birth, intra uterine fetal death and other complications, however, most of the mothers initiate ANC late [1, 2].

Maternal death is a major problem and it is still high. Globally, 830 women die each day, and more than 303, 000 women die each year as a consequence of pregnancy and childbirth related complications. Almost all, 99%, of those deaths occur in developing countries: sub-Saharan Africa alone account for about 66%, followed by Southern Asia 22% [3, 4].

Though early initiation of ANC is more advantageous in the early detection and management of problems related to pregnancy and preventing adverse complications during pregnancy, women are still starting ANC visit late in their pregnancy. Studies conducted to assess the timing of first ANC visit and its associated factors demonstrated that most of the pregnant mothers initiate ANC visit late that is after16 weeks of GA [5–9].

Woman’s residence, age, educational status, employment status, parity, intention to get pregnant, economic status, health insurance, and traveling time are among the most cited factors which are related to late ANC visit [10].

Although timely initiation of ANC is essential, mothers initiate their ANC late and no more attention is given to the timing of first ANC and its attributes remains not well known. Thus, the study was conducted to assess the factors associated with the timing of ANC attendance in Axum in which studies regarding this issue are lacking. Though, there are some existing studies in Ethiopia and in Tigray as well, studies in the study area, Axum town remain limited. So, to fulfill this gap conducting such a study was essential.

## Methods

### Study area and period

This study was conducted in public health institutions of Axum town from February 1, 2017 to March 30, 2017. Axum is one of the zonal towns in Tigray region which is 1010 km far from Addis Ababa, the capital city of Ethiopia. It is one of the tourist areas in North Ethiopia and an ancient city of great historical and religious significance. According to the 2009 population estimation, the total population of Axum town was estimated to be 54,139 and 12,712 of them were women of reproductive age group.

### Study design

Institutional based cross-sectional study design with quantitative and qualitative approaches.

### Source population

All pregnant women who visited public ANC clinics in Axum town.

### Study population

#### Quantitative part

All pregnant women who visited ANC clinics of the public health facilities in Axum during the data collection period.

#### Qualitative part

All purposively selected pregnant women visiting public ANC clinics in Axum during the data collection period who did not participate in the quantitative part, health care providers working in the ANC department of the health facilities, health extension workers and women’s development army leaders in Axum town.

### Sample size determination and sampling procedure

#### Sample size determination

##### Quantitative part

The sample size for this study was calculated using the formula for a single population proportion considering the following assumptions: 95% confidence interval, Margin of error = 0.05, Proportion (P): 35.4% [11] and 10% was added for non-response rate. Finally, the sample size was found to be 386.

##### Qualitative part

Four health care providers, four health extension workers, four women’s development army leaders and six pregnant mothers were included in the in-depth interview. Eligible women who were critically ill, who were in labor pain and mothers participated in the qualitative part were excluded from interview in the quantitative part.

### Sampling procedure

#### Quantitative part

There were four public health institutions providing ANC services in Axum, two health centres one general hospital and one referral hospital. All of these facilities were included in the study. Pre-survey assessment was carried out to determine the average daily flow of mothers receiving the service in those health institutions. According to the expected number of women in the specified period of data collection, the sample size was proportionally allocated to those health institutions. Finally, individual study subjects were selected from each facility by using systematic sampling techniques.

##### For the qualitative part

The in-depth interviewees (health care providers, health extension workers, pregnant mothers and women’s development army) were selected purposively. Four health care providers (one from each health institution), four health extension workers (one from each health institution), four women’s development army (one from each health institution) and six pregnant mothers, two mothers from the general hospital and four mothers from the remaining health institutions were participated in the in-depth interview. The sample size for the IDI was determined by saturation of ideas.

### Data collection instruments and procedures

#### Quantitative data

Structured interviewer administered questionnaire adapted from different literatures was used to collect the data. Four qualified diploma midwife data collectors were recruited to fill the tools, and one midwife having masters’ degree in clinical midwifery was recruited to supervise the data collection process. Two days training was given for both the data collectors and the supervisor who participated in the pre-test and data collection. After the pre-test was conducted, uncertainties and ambiguities which were found in the questionnaire had been discussed. Finally, the questionnaire was corrected upon completion of the pre-test. The principal investigator and the supervisor were strictly followed and supervised the data collection on daily basis. Data coding and data entry was checked throughout the work. Data cleaning was conducted at the end of data entry.

#### Qualitative data

In-depth interviews were conducted by experts who were experienced with qualitative data collection using an open ended interview guide, and the data were recorded by tape recorder to avoid remembrance problems.

### Operational definitions

#### Antenatal care

Pregnancy related services given by skilled health care providers starting from conception up to the onset of labor including monitoring of the health of the woman and the fetus.

#### Timing of ANC attendance

The first time by which pregnant mothers come to ante natal clinics to get care from health care professionals.

#### Timely ANC initiation

The first ANC visit before 16 weeks of gestational age.

#### Late ANC timing

First ANC visit started at or after 16 weeks of gestational age.

#### Knowledge about ANC

Those participants who scored greater or equal to the mean score from the knowledge measuring questions were considered as having good knowledge about ANC and those participants who scored below the mean score from the knowledge measuring questions were considered as having poor knowledge about ANC [12].

#### Perceived timing of ANC

Those mothers considering that timing of first ANC attendance to be before 16 weeks of GA were considered having appropriate perceived timing of ANC and if they consider that it is after 16 weeks of GA they were considered as in appropriate.

### Data analysis

#### Quantitative part

Data entry and cleaning was done using Epi info and the data was analysed using SPSS version 22. Descriptive data analysis was done to describe the variables under study. Multivariable logistic regression analysis was done to see the independent effect of each variable on the outcome variable. Variables with *p*-value < 0.2 in the bi-variate analysis were exported to the multivariable analysis. Multi-co-linearity was checked using the variance inflation factor (VIF), and those with VIF greater than 10 were excluded from the model. Results were presented using texts, tables, graphs and charts. Adjusted Odds Ratio (AOR) with its 95% Confidence Interval (CI) was used to measure the association between the outcome and independent variables. Significance was declared at *p*-value less than 0.05.

The qualitative tape-recorded data was listened and transcribed, and was quoted and coded using Atlas TI 7.5.4 software. Then major findings were narrated and summarized based on thematic areas and finally triangulated with the quantitative findings.

### Ethical consideration

Ethical clearance was obtained from Mekelle University College of Health Sciences Institution Review Board (IRB). Support letter was obtained from Tigray Regional Health Bureau (TRHB) and Axum town health Bureau and respective health institution before field activities started. Written informed consent was obtained from the study subjects after explaining the study objectives and procedures. The participant’s personal identification was not included in the study questionnaire to maintain anonymity. Confidentiality was maintained throughout the study.

## Results

### Socio demographic characteristics of respondents

All the 386 pregnant women were participated in this study making the response rate of 100%. One hundred forty-six (37.8%) of the study participants were in the age group of 25–29 years and the mean age (+ SD) of the participants was 26.09 (+ 4.65) years ranging from 18 to 42. Ninety four percent of the participants were married. Three hundred and thirty-eight (87.6%) of the participants were from the urban area, and 205 (53.1%) were housewives. Regarding educational status, 163 (42.2%) of the participants took secondary and preparatory education, and the mean monthly income of the respondents was 3360.20 Ethiopian birr (Table 1).

**Table 1:** Socio-demographic characteristics of the pregnant women who visit public health institutions for ANC in Axum town by timing of ANC attendance, Axum, Tigray, Northern Ethiopia, June 2017 (*n* = 386)

Variables		Timely bookers’ *n* = 106(%)	Late bookers’ *n* = 280(%)	Total n (%)
Age in years	18–19	6 (33.3)	12 (66.7)	18 (4.7)
	20–24	31 (23.8)	99 (76.2)	130 (33.7)
	25–29	44 (30.1)	102 (69.9)	146 (37.8)
	30–34	17 (25.8)	49 (74.2)	66 (17.1)
	≥35	8 (30.8)	18 (69.2)	26 (6.7)
Religion	Orthodox	100 (28.1)	256 (71.9)	356 (92.2)
	Muslim	6 (20)	24 (80)	30 (7.8)
Marital status	In marriage	104 (28.7)	258 (71.3)	362 (93.8)
	Out of marriage	2 (8.3)	22 (91.7)	24 (6.2)
Residence	Urban	96 (28.4)	242 (71.6)	338 (87.6)
	Rural	10 (20.8)	38 (79.2)	48 (12.4)
Educational status	No formal education	7 (17.5)	33 (82.5)	40 (10.4)
	Primary (1–8)	16 (18.4)	71 (81.6)	87 (22.5)
	9–12	37 (22.7)	126 (77.3)	163 (42.2)
	College level and above	46 (47.9)	50 (52.1)	96 (24.9)
Occupation	Employed (Gov’t &NGO)	37 (44)	47 (56)	84 (21.8)
	Self- employee	16 (23.9)	51 (76.1)	67 (17.4)
	House wife	48 (23.4)	157 (76.6)	205 (53.1)
	Farmer	5 (16.7)	25 (83.3)	30 (7.8)
Husband education	No formal education	0 (0)	17 (100)	17 (4.4)
	Primary (1–8)	22 (21.8)	79 (78.2)	101 (26.2)
	9–12	29 (25.7)	84 (74.3)	113 (29.3)
	College level and above	50 (35.5)	100 (64.5)	155 (40.2)
Husband Occupation	Employed (Gov’t &NGO)	56 (37.1)	95 (62.9)	151 (39.1)
	Self- employee	42 (21.5)	153 (78.5)	195 (50.5)
	Farmer	8 (20)	32 (80)	40 (10.4)
Transportation use	Yes	75 (28.7)	186 (71.3)	261 (67.6)
	No	31 (24.5)	94 (75.2)	125 (32.4)
Transportation cost *n* = 261	< 3 birr	8 (22.9)	27 (77.1)	35 (13.4)
	3–9 birr	38 (25.5)	111 (74.5)	149 (57.1)
	> 9 birr	29 (37.7)	48 (62.3)	77 (29.5)

### Significant others related factors

Out of the total study participants, 143 (37%) had advised to start ANC and 41.3% of them have got the advice from health professionals. About 101 (70.6%) of them were informed on when to start their ANC and 57.4% of them were informed to attend ANC before 16 weeks after cessation of their menses (Table 2).

**Table 2:** Significant others related variables of pregnant women who visit public health institutions for ANC in Axum town, by timing of ANC attendance, Axum, Tigray, Northern Ethiopia, June 2017 (*n* = 386)

Variables		Timely bookers *n* = 106(%)	Late bookers’ *n* = 280(%)	Total n (%)
Advised to start ANC	Yes	49 (34.3)	94 (65.7)	143 (37)
	No	57 (23.5)	186 (76.5)	243 (63)
Source of Advice *n* = 143	From Husband	23 (41.1)	33 (58.9)	56 (42.7)
	From HEWs	17 (28.8)	42 (71.2)	59 (41.3)
	From Husband, family, HEWs and friends	9 (32.1)	19 (67.9)	28 (16.1)
Informed when to start *n* = 143	Yes	37 (36.6)	64 (63.4)	101 (70.6)
	No	12 (28.6)	30 (71.4)	42 (29.4)
Informed time to start ANC *n* = 101	before 16 weeks after cessation of menses	33 (56.9)	25 (43.1)	58 (57.4)
	16 weeks after cessation of menses	38 (88.4)	5 (11.6)	43 (42.6)
Decision for ANC initiation	With Husband	70 (30.2)	162 (69.8)	232 (60.1)
	My self	36 (23.4)	118 (76.6)	154 (39.9)
Husband’s courage on timely ANC initiation *n* = 386	Yes	100 (29.2)	244 (70.9)	344 (89.1)
	No	6 (14.3)	36 (85.7)	42 (0.9)
Husband’s courage on timely ANC initiation *n* = 386	Yes	100 (29.2)	244 (70.9)	344 (89.1)
	No	6 (14.3)	36 (85.7)	42 (0.9)
Reason for husband’s not participating on timely ANC initiation *n* = 42	Because pregnancy is normal	5 (22.7)	17 (77.3)	22 (52.4)
	Doesn’t know the occurrence of pregnancy	6 (30)	14 (70)	20 (47.6)
Reason for husband’s not participating on timely ANC initiation *n* = 42	Because pregnancy is normal	5 (22.7)	17 (77.3)	22 (52.4)
	Doesn’t know the occurrence of pregnancy	6 (30)	14 (70)	20 (47.6)

### Obstetric history, current and previous pregnancy related factors of respondents

Among the total participants, 228 (59.1%) were multigravid, and 237 (61.4%) were multiparous. Sixty-five (16.8%) of the respondents were having a problem in their current pregnancy, and 282 (73.1%) of the respondents have planned pregnancy. One hundred and eighty (46.6%) of the respondents recognized that they were pregnant by missing of their menses once and 170 (44%) of the respondents recognized their pregnancy by a laboratory test (Table 3).

**Table 3:** Obstetric and current and previous pregnancy related factors of the pregnant women who visit public health institutions for ANC in Axum town by timing of ANC attendance, Axum, Tigray, Northern Ethiopia, June 2017 (n = 386)

Variables		Timely bookers *n* = 106(%)	Late bookers *n* = 280 (%)	Total n (%)
Gravidity	Primi gravid	35 (26.3)	98 (73.7)	133 (34.5)
	Multi gravid	67 (29.4)	161 (70.6)	228 (59)
	Grand multi gravid	5 (20)	20 (80)	25 (6.5)
History of abortion	Yes	17 (30.4)	39 (69.6)	56 (14.5)
	No	89 (27.0)	241 (73)	330 (85.5)
Type of abortion *n* = 56	Spontaneous	14 (29.2)	34 (70.8)	48 (85.7)
	Induced	3 (37.5)	5 (62.5)	8 (14.3)
Parity	Nulli parous	38 (25.5)	111 (74.5)	149 (38.6)
	Multi parous	68 (28.7)	169 (71.3)	237 (61.4)
Number of alive children *n* = 237	1–2	58 (31.7)	125 (68.3)	183 (79.6)
	> 2	40 (85.1)	7 (14.9)	47 (20.4)
History of child death *n* = 237	Yes	2 (25)	6 (75)	8 (3.4)
	No	66 (28.8)	163 (71.2)	229 (96.6)
History of still birth *n* = 237	Yes	4 (50)	4 (50)	8 (3.4)
	No	64 (27.9)	165 (72.1)	229 (96.6)
Complication in previous delivery *n* = 237	Yes	24 (39.3)	37 (60.7)	61 (25.7)
	No	44 (25.0)	132 (75.0)	176 (74.3)
Intended pregnancy	Yes	92 (32.6)	190 (67.4)	282 (73.1)
	No	14 (13.5)	90 (86.5)	104 (26.9)
Problems/illness in current pregnancy	Yes	32 (49.2)	33 (50.8)	65 (16.8)
	No	74 (23.1)	247 (76.9)	321 (83.2)
Means of pregnancy recognition	While menses is missed	45 (25)	135 (75)	180 (46.6)
	Due to physiological changes	9 (25)	27 (75)	36 (9.3)
	Urine test	52 (30.6)	118 (69.4)	170 (44)
Type of complication in previous delivery *n* = 61	Anemia	1 (100)	0 (0)	1 (1.6)
	APH	1 (33.3)	2 (66.7)	3 (4.9)
	C/S	8 (32)	17 (68)	25 (41)
	PIH	5 (50)	5 (50)	10 (16.4)
	Hyper emesis gravidarum	1 (14.3)	6 (85.7)	7 (11.5)
	Polyhydramnios	1 (50)	1 (50)	2 (3.3)
	Spinal bifida	1 (50)	1 (50)	2 (3.3)
	Still birth	4 (50)	4 (50)	8 (13.1)
	Tear	1 (33.3)	2 (66.7)	3 (4.9)

### Previous ANC service utilization related factors of respondents

Out of the total 386 respondents, 237 (61.4%) pregnant mothers had experienced ANC service utilization in their previous pregnancies and 43.5% of them were experienced the ANC for their first pregnancy. About 153 (64.6%) of them started the ANC in their previous pregnancies after 16 weeks of gestational age (Table 4).

**Table 4:** Previous ANC service utilization related factors of pregnant women who visit public health institutions for ANC in Axum town by timing of first ANC attendance, Axum, Tigray, Northern Ethiopia, June 2017 (*n* = 386)

Variables		Timely bookers *n* = 106 (%)	Late bookers *n* = 280 (%)	Total n (%)
History of previous ANC utilization	Yes	68 (28.7)	169 (71.3)	237 (61.4)
	No	38 (25.5)	111 (74.5)	149 (38.6)
For which pregnancy was the ANC experience? *n* = 237	1st	37 (35.9)	66 (64.1)	103 (43.5)
	1st &2nd	23 (27.7)	60 (72.3)	83 (35)
	1st- 3rd	6 (17.1)	29 (82.9)	35 (14.8)
	1st-4th	2 (12.5)	14 (87.5)	16 (6.8)
Previous ANC experience time *n* = 237	before 16 weeks of gestation	23 (29.9)	54 (70.1)	77 (32.5)
	after 16 weeks of gestation	45 (28.1)	115 (71.9)	160 (67.5)
Do you think that ANC service utilization can be hindered by waiting time *n* = 237	Yes	25 (28.1)	64 (71.9)	89 (37.6)
	No	43 (29.1)	105 (70.9)	148 (62.4)
Maximum time spent for previous first visit ANC *n* = 237	< 120 min	17 (32.1)	36 (67.9)	53 (22.4)
	120–240 min	36 (26.7)	99 (73.3)	135 (57.0)
	> 240 min	15 (30.6)	34 (69.4)	49 (20.7)
Maximum time spent for previous repeated ANC visits *n* = 237	< 120 min	35 (25.4)	103 (74.6)	138 (58.2)
	120–240 min	33 (33.3)	66 (66.7)	99 (41.8)

### Respondents’ knowledge about ANC and perception on timing of ANC

Regarding respondents’ knowledge, 224(58%) of the respondents have good knowledge about ANC and the importance of ANC. More than half (57.5%) of the participants perceived ANC timing to be after 16 weeks of gestational age.

### The timing of ANC attendance

**Fig. 1:**
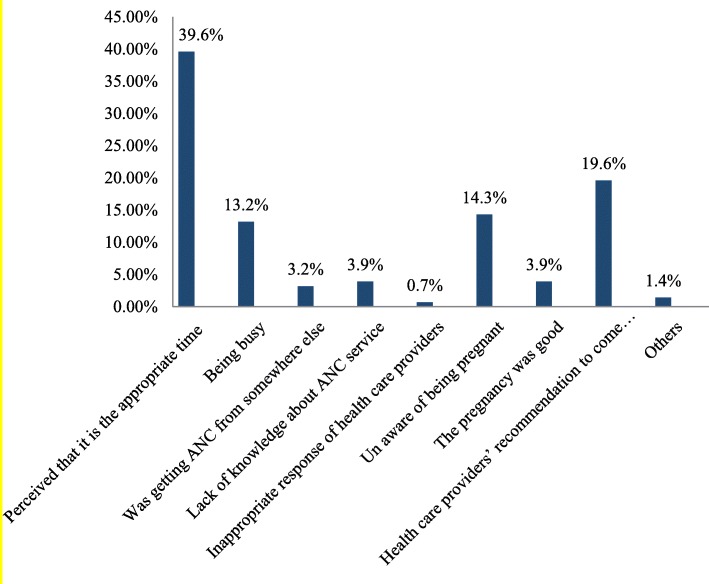
Reasons for late ANC attendance mentioned by the pregnant women who visited public health institutions for ANC in Axum town, Tigray, Northern Ethiopia, 2017

The information on timing of ANC attendance was gathered from mothers’ recall. If they fail to remember, it was extracted from their medical charts. The magnitude of timely initiation of ANC was 27.5% (95% CI: 23–32%). But, nearly three fourth of the respondents (72.5%) did not book their ANC visit timely. The mean gestational age (±SD) at first ANC attendance of respondents’ was 18.49 ± 5.548 weeks. The timing of first ANC attendance was ranged from 4 weeks up to 38 weeks of gestational age. Around 40% of the respondents who started their ANC visit late reported perceiving as it is appropriate time as the reason for their late ANC attendance and 55 (19.6%) of the late booker respondents reported that health care providers’ recommendation to come for first ANC follow up after the fetus starts movement as a reason for their late booking (Fig. 1).

Results of the qualitative study have also similar findings. The IDI interviewees reported that there is a problem with health care providers; they recommend mothers to come for their first ANC follow up after 16 weeks of gestational age.

A 25 years old gravida II para I pregnant mother who came for first ANC visit to the hospital at 24 weeks of gestational age said “*ANC should be started early in the pregnancy at 2, 3 months. But we do not make it practical because of several reasons. For example, I came while I miss my menses once, but the health care provider checked that I am pregnant and told me to come after the fetus starts to move. Then, now as you see me, I came at 6 months of my pregnancy after the baby starts a movement as their recommendation*”.

The major reasons of late ANC attendance reported by the mothers who were late for their ANC visit were perceiving that as it is correct time (39.6%), due to health care providers recommendation to come after 16 weeks of GA (19.6%) and unaware of being pregnant (14.3%).

### Factors associated with timing of ANC attendance

On multivariable analysis, maternal educational status, maternal knowledge about ANC and importance of ANC, perceived timing of ANC, unintended pregnancy, advice from significant others and problem in current pregnancy were found to be the independent factors of late timing of ANC attendance (Table 5).

**Table 5:** Logistic regression of factors that influence timing of ANC attendance of the respondents in Axum town, Tigray, Northern Ethiopia, June 2017 (*n* = 386)

Variables		Timing of ANC			COR with 95% CI	AOR with 95% CI
		Timely Bookers *n* = 106(%))	Late Bookers *n* = 280 (%))	Total n (%)	COR (CI)	AOR (CI)
Maternal education	Primary and below	23 (18)	105 (82)	128 (33.2)	1	1
	Secondary and above	83 (32.2)	175 (67.8)	258 (66.8)	2.165 (1.285, 3.647)	**2.619 (1.216, 5.642)**
Intended pregnancy	Yes	92 (32.6)	190 (67.4)	282 (73.1)	3.113 (1.682, 5.761)	**2.873 (1.231, 6.707)**
	No	14 (13.5)	90 (86.5)	104 (26.9)	1	1
Complication in previous pregnancy *n* = 237	Yes	24 (39.3)	37 (60.7)	61 (25.7)	1.946 (1.050,3.605)	1.756(.810, 3.807)
	No	44 (25.0)	132 (75.0)	176 (74.3)	1	1
Problem in current pregnancy	Yes	32 (49.2)	33 (50.8)	65 (16.8)	3.237 (1.865, 5.617)	**3.596 (1.525, 8.481)**
	No	74 (23.1)	247 (76.9)	321 (83.2)	1	1
Advice from significant others	Yes	49 (34.3)	94 (65.7)	143 (37)	1.701 (1.079, 2.682)	**2. 333 (1.101, 4.945)**
	No	57 (23.5)	186 (76.5)	243 (63)	1	1
Perceived time to start ANC	Appropriate	61 (37.2)	103 (62.8)	164 (42.5)	2.329 (1.477, 3.673)	**3.445 (1.611, 7.364)**
	In appropriate	45 (20.3)	177 (79.7)	222 (57.5)	1	1
Number of alive children *n* = 230	1–2	58 (31.7)	125 (68.3)	183 (79.6)	2.651 (1.121, 6.274	1.367 (.503, 3.715)
	> 2	7 (14.9)	40 (85.1)	47 (20.4)	1	1
Maternal knowledge	Good	71 (31.7)	153 (68.3)	224 (58.0)	1.684 (1.054, 2.689)	**2.748 (1.074, 7.034)**
	Poor	35 (21.6)	127 (78.4)	162 (42.0)	1	1
Decision of ANC initiation	With husband	70 (30.2)	162 (69.8)	232 (60.1)	1.416 (.888, 2.259)	.887 (.406, 1.939)
	My self	36 (23.4)	118 (76.6)	154 (39.9)	1	1
Did your husband encourage you to attend ANC timely?	Yes	100 (29.2)	244 (70.9)	344 (89.1)	2.459 (1.005, 6.018)	.629 (.171, 2.314)
	No	6 (14.3)	36 (85.7)	42 (10.9)	1	1

*1 = Reference*

Mothers whose educational status was secondary school and above were about two times more likely to attend ANC timely compared to the mothers with primary education or less (AOR: 2.619, 95% CI,1.216–5.642).

This is corroborated by the qualitative findings. The participants in the IDI said that the educational status of mothers has an effect on ANC initiation time. One health care provider said that “*... Educating mothers using different methods is necessary. Because, there are a lot of mothers who are not educated, who might not go to anywhere out of the house and become shy and afraid while they come to our health facility”.*

The odds of pregnant women having good knowledge about the importance of ANC and early ANC booking to book timely was three times (AOR: 2.748, 95% CI, 1.074–7.034) higher compared to those pregnant mothers who have poor knowledge about the importance of ANC and early ANC booking. The qualitative findings strongly support this idea.

Respondents who got pregnant intentionally were about three times more likely to attend ANC timely compared to those who got pregnant unintentionally (AOR: 2.873, 95% CI, 1.231–6.707).

In a similar manner, those respondents who were informed to start ANC were about twice more likely to book timely than those who were not informed (AOR: 2.333, 95% CI, 1.101–4.945).

Those participants who were having any problem in their current pregnancy were about three times more likely to begin their ANC timely compared to those who did not have any problem (AOR: 3.596, 95% CI, 1.525–8.481) and those mothers who perceived that the correct timing of ANC initiation is before 16 weeks of gestational age were three times more likely to book their ANC timely than those who perceived it inappropriately (AOR: 3.445, 95% CI, 1.611–7.364).

## Discussion

The overall magnitude of timely attendance of ANC was 27.5% (95% CI: 23%-32. This finding was almost similar with the studies done in Benin (24.6%) [13], Tanzania (29%) [14], Uganda (27.9%) [15], Halaba Kulito (27.1%) [16] and Debre birhan (26.2%) [17].

Finding of this study is higher compared to studies done in, Zambia (17%) [18], Nigeria (15.4%) [19], Tanzania (12.4%) [20], Ambo (13.2%) [7] and Arba Minch (17.4%) [21]. This might be due to the difference in socio-demographic characteristics of respondents, infrastructures and time disparity and due to the more efforts done recently to decrease maternal mortality.

This finding was lower as compared to similar studies conducted in Gondar (35.4%) [11] and Mekelle (32.7%) [22] and this might be due to differences in socio-demographic characteristics of study participants, infrastructures and accessibility of health facilities.

The mean GA at first ANC booking of this study was 18.49 weeks with a SD of 5.548 (18.49 ± 5.548 weeks) weeks and it was lower compared to the studies conducted in Uganda which were 27.9 weeks [15], Nigeria (20.86 ± 6.39 weeks) [19], Arba Minch (5 ± 1.5 months) [21], and this might be due to difference in living standards, time variation. It was almost nearly comparable with the studies in Ambo (4.7 months) [7] and Gondar (17.7 ± 7.5 weeks) [11].

In the multivariable analysis, maternal education was found to be significantly associated with timely attendance of ANC; mothers whose educational status was secondary school and above were two times more likely to attend their ANC timely compared to the mothers who took primary school or who had no formal education. This study is similar to the studies conducted in Vietnam, Benin, Uganda, Ambo and Adigrat [6, 7, 13, 23, 24]. This might be due to, educated mothers might be knowledgeable about what is necessary during pregnancy, the importance of ANC and early booking and they might book timely.

Maternal level of knowledge was found to be significantly associated with timely beginning of ANC; respondents who had good knowledge about the importance of ANC and early booking were about three times more likely to book timely as compared to those respondents who had poor knowledge about the importance of ANC and early booking. This is equivalent with the study findings in Benin, Zambia, Tanzania, Rwanda, Ambo, Debre brhan, Mekelle and Adigrat in which mothers who lack knowledge about ANC were more likely to book late [6–8, 13, 17, 20, 22, 25].

Unintended pregnancy was also found to be the predictor of late ANC commencement. The likelihood of pregnant women with planned pregnancy to book timely was about three times higher compared to those who have the unplanned pregnancy. This was in line with the studies conducted in different areas in which mothers with the unplanned pregnancy were more likely to book late; Zambia, Arba Minch, Addis Ababa, Debre brhan and Adigrat [6, 8, 17, 21, 26]. This might be due to: if mothers did not plan to get pregnant, they might not know whether they are pregnant or not timely and they will be late for first ANC booking and also even if they know that they are pregnant early, they might not be interested with the pregnancy, they might be careless for that pregnancy and they fail to book timely.

This study also showed advice from significant others to be a significant factor for timing of ANC booking; those respondents who were informed to start ANC were about twice more likely to book timely than those who were not informed and it is in line with the studies done in Arba Minch, Addis Ababa and Gondar in which the participants who were not advised to start ANC before they start were more likely to book late [11, 21, 26]. The pregnant mothers may not know the correct timing of ANC. Advising mothers regarding timing of ANC attendance might motivate them to start their ANC timely; if they were informed on the time when to start, they might begin at the time they were advised to start.

Having any problem in current pregnancy was also found to be a factor for timely booking. Those participants who had any problem in their current pregnancy were about three times more likely to begin their ANC timely compared to those who did not have any problem and it is comparable with the study results from Ghana, Malawi and Kenya, Benin and Mekelle [13, 22, 27]. This might be because of antenatal care is perceived by the mothers as curative rather than the preventive measure that is why the mothers start ANC timely if they get any pregnancy related illness.

It is also found that perceived timing of ANC to be significantly associated with ANC attendance; Those mothers who perceived that correct timing of ANC initiation is before 16 weeks of gestational age were 3 times more likely to book their ANC timely than those who perceived it inappropriately ANC and this was supported by the studies conducted in Benin, Addis Ababa, Gondar, Mekelle and Adigrat [6, 11, 13, 22, 26]. This might be because of, mothers think that the correct timing of ANC attendance is after the pregnancy is physically known by the family and health care providers, most probably after 16 weeks of GA and they believe that as they get adequate ANC services starting that time.

This study has also attempted to assess if mothers’ previous experience of timely ANC attendance has an effect on the early timing of ANC in their current pregnancy. But, previous ANC service utilization was not found to be statistically associated with timing of ANC; the pregnant mothers who experienced early timing of ANC in their previous pregnancy preceding the current pregnancy were failed to attend timely for the current pregnancy OR (95% CI): 1.088 (0.599–1.978)) and this is similar with study findings in Addis Ababa and Mekelle [22, 26]. This might be due to mothers’ perception of not benefited from their previous early timing of ANC.

This study was carried out using the combination of qualitative and quantitative methods; to dig out reliable information and strengthened the quantitative findings. However, This study was done only on public health facilities, private clinics were not included in this study; that is possible differences between the mothers who attend ANC in public health institutions and those who attend in private clinics was not observed. In addition, as this study is cross sectional study, the time of occurrence of the cause and the effect might not be known, we cannot know whether the cause antedated the effect.

## Conclusion

This study found high prevalence of delayed timing of ANC attendance in Axum town attributed to lack of awareness on the importance of ANC and appropriate timing of ANC; health care providers’ recommendation to come after 16 weeks of gestation or after the fetus starts movement, unaware of being pregnant, and being busy. In addition, respondents low level of education, poor knowledge about ANC and its importance, having unintended pregnancy, not getting any problem in current pregnancy, lack of advice about timing of ANC and wrong perceived timing of ANC were significantly associated with increased odds of delayed timing of ANC attendance.

Therefore, efforts should be done to increase the knowledge of mothers, and front line health care providers about importance of ANC and timing of ANC booking.

## Data Availability

The datasets during and/or analyzed during the current study is available from the corresponding author on reasonable request.

## Abbreviations

ANC: Ante Natal Care
AOR: Adjusted Odds Ratio
APH: Ante Partum Hemorrhage
C/S: Caesarean Section
CI: Confidence Interval
COR: Crude Odds Ratio
EDD: Expected Date of Delivery
EDHS: Ethiopian Demographic Health Survey
GA: Gestational Age
HEWs: Health Extension Workers
IDI: In depth Interview
MMR: Maternal Mortality Ratio
OR: Odds Ratio
PIH: Pregnancy Induced Hypertension
SD: Standard Deviation
SPSS: Statistical Package for Social Sciences
TRHB: Tigray Regional Health Bureau
UNFPA: United Nations Population Fund
UNICEF: United Nation Children’s Fund
VIF: Variance Inflation Factor
WHO: World Health Organization
